# Can HIV‐positive gay men become parents? How men living with HIV and HIV clinicians talk about the possibility of having children

**DOI:** 10.1111/1467-9566.13218

**Published:** 2020-11-22

**Authors:** Robert Pralat, Fiona Burns, Jane Anderson, Tristan J. Barber

**Affiliations:** ^1^ Department of Sociology University of Cambridge Cambridge UK; ^2^ Institute for Global Health University College London London UK; ^3^ Royal Free London NHS Foundation Trust London UK; ^4^ Homerton University Hospital NHS Foundation Trust London UK

**Keywords:** gay men, health communication, HIV transmission, reproductive health, risk, sexual health, sperm washing, U = U, undetectable

## Abstract

It is now established that people living with HIV who have an undetectable viral load and adhere to antiretroviral treatment cannot transmit HIV to their sexual partners. Previous research has shown that ‘being undetectable’ changes how HIV‐positive gay men experience their sex lives. But how does it affect gay men’s reproductive behaviours? And what influence does it have on views about parenthood at a time when gay fatherhood has become more socially accepted and publicly visible? Drawing on qualitative interviews with patients and clinicians at four HIV clinics in London, we identify differences in how interviewees talked about the possibility of having children for HIV‐positive men. Both groups, unprompted, frequently referred to sperm washing as a method enabling safe conception. However, whereas clinicians talked about sperm washing as an historical technique, which is no longer necessary, patients spoke of it as a current tool. The men rarely mentioned being undetectable as relevant to parenthood and, when prompted, some said that they did not fully understand the mechanics of HIV transmission. Our findings offer new insights into how biomedical knowledge is incorporated into people’s understandings of living with HIV, raising important questions about how the meanings of being undetectable are communicated.

## 1 INTRODUCTION

It is now established that people living with HIV who have an undetectable viral load and adhere to antiretroviral treatment cannot transmit HIV to their sexual partners. The dominant clinical position has shifted from the recognition that having an undetectable viral load *significantly reduces* the risk of HIV transmission to the more recent acknowledgement that, for people living with HIV whose viral load is undetectable, there is effectively *no risk* of transmitting HIV to a sexual partner (Cohen 2019, Rodger *et al*. 2019, The Lancet HIV 2017). This shift is captured in the slogan ‘Undetectable Equals Untransmittable’, or ‘U = U’, launched by Prevention Access Campaign in 2016. There is growing evidence of how the awareness of ‘being undetectable’ affects gay men’s sex lives (Bourne *et al*. 2016, Grace *et al*. 2015, Holt *et al*. 2015). But little is known about how it affects other aspects of male same‐sex intimacy – in particular, how it influences thinking about parenthood.

In this article, we present findings of a qualitative interview study conducted at four HIV clinics in London between May and December 2016, shortly before U = U received official endorsement from major medical and public health organisations including the British HIV Association and the US Centers for Disease Control and Prevention, but when ‘undetectable’ had already become a common category used by people living with HIV (Persson 2016, Race 2015, Young *et al*. 2019). The study explored the issue of parenthood, and gay fatherhood in particular, in the context of intimate relationships and healthcare provision, through interviews with HIV clinicians and younger (20–45 years old), predominantly gay‐identified men living with HIV who did not have children.^1^ This article focuses specifically on (1) how the possibility of having children for HIV‐positive men is perceived by gay men living with HIV and by HIV clinicians, and (2) how advances in medical science are incorporated into perceptions of parenthood as a possibility. In the context of growing social acceptance and public visibility of gay fatherhood, we argue for moving beyond strictly *sexual* understandings of viral undetectability to include how being undetectable is understood in relation to non‐sexual aspects of same‐sex intimacy, including *reproduction*. In doing so, we encourage finding ways to effectively communicate about the implications of being undetectable which could guide considerations of gay, bisexual and other men who have sex with men about the possibility of having children, and foster a more holistic understanding of HIV transmission.

Before presenting our findings, we situate our study within the social science and public health literature on HIV, showing how advances in medicine coupled with cultural changes have influenced HIV‐positive people’s experiences of sexual and reproductive relationships.

## 2 HIV, SEXUALITY AND REPRODUCTION

As a result of advancements in highly active antiretroviral therapy, people living with HIV in countries with access to treatment, such as the UK, have near‐normal life expectancy (May *et al*. 2014). Despite the persistence of HIV stigma (Jaspal and Nerlich 2020, Rai *et al*. 2018, Walker 2019), living with HIV has become in many ways ‘normalised’ (Mazanderani and Paparini 2015, Moyer and Hardon 2014, Persson 2013), which manifests, among other things, in conventional expectations about the future. Antiretroviral treatment not only improves health outcomes but also reduces potential infectivity – a phenomenon described in public health as ‘treatment as prevention’ or TasP. There is now a consensus among scientists and clinicians that effective antiretroviral treatment, which lowers the viral load to ‘undetectable’ levels, eliminates the risk of HIV transmission to sexual partners (Cohen 2019, The Lancet HIV 2017).^2^ This marks a shift from the recognition that having an undetectable viral load *significantly reduces* the risk of transmission – a dominant view among medical experts until 2017 – to the current acknowledgement that there is effectively *zero risk* of transmission (Rodger *et al*. 2019). In other words, to use a slogan from Terrence Higgins Trust’s 2019 campaign, people on effective HIV treatment *‘can’t pass it on’*.

There is a sizeable amount of research documenting the experiences of people living with HIV in what some scholars have referred to as the ‘TasP era’ (Young *et al*. 2019). Much of this work pays specific attention to how people incorporate biomedical knowledge into their everyday understandings of living with HIV, including intimate practices, especially sexual decision making (Bourne *et al*. 2016, Grace *et al*. 2015, Persson 2016). For example, in an Australian study of serodiscordant relationships – where one partner is HIV‐positive and the other HIV‐negative – Persson (2016) draws attention to the centrality of undetectability in people’s narratives of sex. She notes that ‘“undetectable” was invariably deployed as shorthand for the safety of serodiscordant sex, no matter what a couple’s sexual activities’ (387). In recent research conducted with communities most affected by HIV in Scotland, Young *et al*. (2019) report that most participants described themselves, unprompted, as undetectable, which shaped how they talked about their embodied experiences of living with HIV. The authors describe the articulations of being undetectable as expressions of ‘biosocial HIV identities’.

The changing reality of living with HIV has significant implications for the experience of, and decisions about, not only partnering but also parenting. Currently, the rate of vertical transmission in the UK is at an all‐time low at 0.27% (Peters *et al*. 2017) and, based on a large‐scale French study, among mothers who start antiretroviral therapy before conception and maintain a suppressed viral load, HIV transmission is ‘virtually zero’ (Mandelbrot *et al*. 2015). As the risk of mother‐to‐child transmission has been largely eliminated, it is easier for clinicians to support HIV‐positive women in their planning for parenthood. Pre‐conception advice has also changed in situations where the man is HIV‐positive and the mother‐to‐be HIV‐negative. Men living with HIV who wanted to conceive with a serodiscordant female partner were historically advised to undergo a procedure known as *sperm washing*, separating spermatozoa (which do not carry HIV) from seminal fluid and associated non‐sperm cells (Nicopoullos *et al*. 2010). However, using this method is expensive, few fertility clinics offer it and many couples have been reluctant to use it as they prefer to conceive ‘naturally’ (Kelly *et al*. 2011, Siegel *et al*. 2018). As TasP has become accepted practice, and especially in light of evidence supporting U = U, clinicians have increasingly endorsed natural conception (Barber *et al*. 2019).

Qualitative studies have shown that, for heterosexual men living with HIV, having an undetectable viral load is a key consideration in managing conception (Kelly *et al*. 2011) and that serodiscordant heterosexual couples rely on the effectiveness of antiretroviral drugs to ensure that HIV is not transmitted to either the sexual partner or the child (Newman *et al*. 2018). Existing research on the meanings of being undetectable among gay men living with HIV (Bourne *et al*. 2016, Grace *et al*. 2015, Race 2015) makes no mention of possible reproductive implications of having an undetectable viral load. It is unclear, however, if this is because of researchers’ explicit focus on gay men’s sex lives or because having children is unimportant to HIV‐positive gay men. While it is likely that few of them decide to become parents, limited evidence suggests that, similar to heterosexual men living with HIV (Rodriguez *et al*. 2017, Siegel *et al*. 2018, Weber *et al*. 2017), it is not uncommon for HIV‐positive gay men to want or plan to have children. In a quantitative study conducted at a London HIV clinic, Sherr (2010) found that approximately one third of men who have sex with men ‘had considered having children’ (5). In a more recent qualitative study conducted in Australia, some HIV‐positive gay men were co‐parenting children conceived in prior heterosexual relationships or through donor conception whereas others considered surrogacy or fostering (Newman *et al*. 2018).

The multiplicity of pathways to gay fatherhood outlined by Newman *et al*. (2018) reflects a transformation in same‐sex intimacy on which HIV research with men who have sex with men remains largely silent. In western countries, increasing numbers of openly gay men are deciding to become parents. Whereas many gay men, especially from older generations, have children from previous heterosexual relationships, more recently there has been growing public visibility and social acceptance of ‘intentional’ gay fathers: that is, gay men who pursue parenthood through routes such as adoption and surrogacy or by providing sperm to – and, in some cases, entering into co‐parenting arrangements with – female friends and other women. There is now a large body of social science literature on gay fatherhood (for more recent studies, see, for example, Blake *et al*. 2017, Dempsey 2012, Murphy 2013, Riggs *et al*. 2015, Smietana *et al*. 2014, Tornello and Patterson 2015). But this literature rarely mentions HIV, except for situating gay‐father families among historical shifts in same‐sex intimacy, including the advent of AIDS in the 1980s (Goldberg 2012, Lewin 2009, Stacey 2006). Gay *and* HIV‐positive parenthood remains invisible, despite the fact that more gay men than ever before are living with HIV, and especially so in urban settings such as London, where the increase in ‘family diversity’ is also most evident.

The lack of crossover between research on gay men living with HIV and studies of gay men pursuing parenthood is, perhaps, surprising when we consider the fact that there are growing numbers of both HIV‐positive gay men and gay fathers (Pralat 2015). Gay fathers living with HIV may not be high in numbers but, as documented by Sherr (2010) and Newman *et al*. (2018), they do exist, while younger HIV‐positive gay men may want to become parents in the future. It is thus timely to ask: how do gay men living with HIV perceive the prospect of having children and their ability to do so? And, in light of the rapidly changing HIV science, what kind of biomedical knowledge do they rely upon when they form their views about parenthood?

## 3 ABOUT THE STUDY

Between May and December 2016, the first author conducted qualitative semi‐structured interviews with 25 patients and 16 healthcare practitioners at four London HIV clinics. We sought to interview HIV‐positive men who were gay or bisexual, 20–45 years old and without children, as well as clinical staff working with this patient group. In order to attract a wider pool of patients – and not only those interested in having children – the study was advertised as research on ‘men’s attitudes to intimate life’. Recruitment for the study was facilitated by local clinical research teams who were advised that it was important for us to reach men with a variety of views about parenthood. Our study did not aim to provide a statistically representative account, but every effort was made to capture views from diverse perspectives. The study was approved by the London – Camberwell St Giles Research Ethics Committee (REC reference: 16/LO/0030) and by the School of Humanities and Social Sciences Research Ethics Committee at the University of Cambridge.

### 3.1 Participants

Men living with HIV were aged between 20 and 45 (the median age was 35); they were born between 1970 and 1995. All but two identified as gay men (one man identified as bisexual and another man refused to identify on the basis of sexuality at the time of the interview). None of the men identified as transgender. Just over half were UK‐born, four came from another European country and eight from outside Europe, including countries in Asia, Oceania, South America and the Caribbean. Overall, the men came from 11 different countries, including the UK. Using the ethnic group categories from the UK census, 17 men were white, five Asian, two mixed‐race (white/black) and one black.

All but two men had, or were studying for, a university degree. Two men were unemployed at the time of the interview, one was a full‐time student and the rest worked in a variety of sectors. Of the 25 men, 13 were single, 11 had a partner and one man was dating. Of the 11 men in a relationship, all had male partners (four were married or in a civil partnership). The length of the men’s relationships ranged from 2 months to 10 years. Three men had a partner who was also HIV‐positive and eight had HIV‐negative partners.

The age at HIV diagnosis ranged from 20 to 34 (the median age was 29), and the time since diagnosis from 1 month to 15 years (the men were diagnosed between 2001 and 2016). All men were on antiretroviral treatment at the time of the interview.

Overall, it proved more difficult to recruit black men, men in their early 20s, men without university education and men who identified as bisexual. Since, of the 25 men, 23 identified as gay, we decided to use the term ‘gay men’ when describing the men as a group, aware of the fact that our data did not allow us to represent bisexual perspectives in a meaningful way.

HIV clinicians included five physicians, five sexual health advisers, three nurses and three psychologists. For some clinicians, men who have sex with men constituted more than 90% of their patients; for others, less than a half. Many clinicians had previously worked in clinics with different patient demographics. Some had worked in HIV medicine since the first AIDS cases were identified in the 1980s; others had begun working in this area more recently.

### 3.2 Interviews

All interviews were conducted and audio‐recorded in private rooms in the clinics where participants had been recruited. Topic guides were used flexibly.

Men living with HIV were first asked about what had made them want to take part in the study. Initial answers led to subsequent questions, which covered, in no specific order: views about parenthood; experiences of intimate and personal relationships, including partners, family of origin and friends; and thoughts about the future. HIV was addressed in relation to these topics based on information interviewees volunteered, usually unprompted. The men were asked about how (and why) their approach to living with HIV had changed over time. Towards the end of the interview, they were asked if they had ever discussed parenthood or reproductive health with HIV clinicians; whether they would like, or would have liked, to discuss this topic; and whether they thought there were any needs for support or information. The average length of patient interview recordings was just over an hour and a half. As a thank you for their time, all patient interviewees were given £25 gift vouchers.

HIV clinicians were first asked about what their job involved and what patient groups they worked with. They were then asked, in no specific order, about contexts in which parenthood or reproductive health was addressed in their work with patients; their experiences of discussing reproduction with men who have sex with men; and their perceptions of how men’s intimate relationships had changed over time and what role HIV had played in these changes. Towards the end of the interview, clinicians were asked about needs for support or information. The average length of practitioner interview recordings was just under an hour.

### 3.3 Data analysis

Once the interviews had been transcribed, the first author managed and analysed the data, anonymising interview transcripts and identifying parts of transcripts that concerned both HIV and parenthood. From this smaller dataset, a number of themes were identified that captured sentiments expressed in multiple interviews.

The starting point for this article was an observation that, in the interviews, both men living with HIV and HIV clinicians often referred to *sperm washing*, even though they were never asked about it directly. However, the two groups of interviewees talked about sperm washing in very different ways. The intriguing discrepancy in patients’ and practitioners’ perceptions of sperm washing prompted a closer inspection of interview extracts which referred to this technique. Since clinicians commented on sperm washing in relation to its redundancy in the context of viral undetectability, interview extracts referring to *being undetectable* were also re‐examined. Ultimately, the observed contrast between patients’ and practitioners’ accounts drew our attention to the ways in which the two groups understood *how* HIV‐positive gay men could become parents with the use of their own sperm and, by extension, what the men understood about HIV transmission. The interview transcripts were finally re‐read in search for further answers to these questions. Data analysis was conducted by the first author who also drafted the article to which other authors contributed.

## 4 FINDINGS

In what follows, we present our findings in three sections. First, we show how, when asked about the possibility of having children, men living with HIV expressed uncertainty about their understanding of HIV transmission, often referring to sperm washing as a means to parenthood. Second, we demonstrate how sperm washing featured in HIV clinicians’ narratives as an historical procedure which is no longer necessary when the man has an undetectable viral load. Third, we show how men’s thinking about parenthood was constrained by concerns about safe conception and how it limited their perceived ability to have biological children. Throughout our analysis, we refer to patient interviewees using pseudonyms and indicating their age and to practitioner interviewees by specifying their profession.

### 4.1 ‘I think there’s the option of doing something to the sperm’

Men in our study expressed a range of parenting desires, which had been shaped in complex ways, had usually changed over time and often seemed contingent or flexible. Overall, 12 men could be described as wanting to become parents in the future, nine were more inclined towards not having children and four were undecided or could not be placed in either category. When asked if they had ever thought about their HIV status in relation to parenthood, some men said that they did not fully understand the risks of HIV transmission:

It’s not something I actually understand 100%. I believe that a mother can be HIV‐positive and carry a child and child not be positive… [But] if they used my sperm in surrogacy to get a woman pregnant, I don’t know if that woman could catch HIV or if the baby could be HIV‐positive. I don’t know. (Ben, aged 33)

I remember thinking [when I was diagnosed with HIV] that I didn’t actually understand how exactly the transmission worked. So I knew a mother can pass it to a daughter – sorry, a mother can pass it to a child – but I didn’t know whether or not the father could pass it to a child… So I just wasn’t sure whether or not it would be possible for me to have my own child, through surrogacy or whatever else. (Liam, aged 27)

Most men assumed that it *was* possible for HIV‐positive men to become fathers without transmitting the virus, but they were unsure about *how* to eliminate or minimise the risk of transmission. As they pondered ways in which transmission could be avoided, their attention focused on sperm. For example, 41‐year‐old Ian commented: ‘I don’t know whether that’s possible if you’re HIV‐positive to actually inseminate someone with your own sperm – whether they can remove HIV from it, I don’t know. I never looked into it, but I’m sure that there are some ways of doing it.’ Owen, aged 23, made a similar remark: ‘I don’t know if there’s an HIV scrubbing that you can sort of do, I don’t know. I certainly don’t know what options are available for HIV‐positive people trying to have kids.’ Also hesitating was 25‐year‐old Blake: ‘I’m trying to think of how you do it… I think there’s the option of doing something to the sperm and then the sperm being given to whoever is having the baby.’ As we can see in these three quotations, the men’s narratives were full of uncertainty: they were punctuated by ‘I don’t knows’ and question marks about ‘available options’. References to ‘removing HIV’, ‘HIV scrubbing’ or ‘doing something to the sperm’ alluded to sperm washing. Some men referred to sperm washing more explicitly, as they recalled how they had found out about it:

I know that if you have HIV – I read somewhere, I don’t know if I’m wrong, it’s just something that comes to my mind – now they know how to, like, clean the sperm from HIV and inseminate. Yes, I’m aware of that. But no more than that. I can’t remember. I read somewhere… I’m not sure. (Juan, aged 40)

It might even have been [when I was diagnosed]. I know someone mentioned, don’t worry, you can still have children – there’s this thing called sperm washing. Someone at least mentioned just about everything I may have had an issue with, so I was at least aware. And then they brought it up again at the [HIV support] group. So I knew it was an option. (William, aged 28)

Two men recalled finding out about sperm washing from the soap opera *EastEnders* – which, across all the interviews, was the only reference to any kind of media portrayal of HIV‐positive parenthood:

I haven’t watched it for years, but in *EastEnders* there was this guy, Mark Fowler, and he had HIV and then he wanted to have this baby. And they sort of came up with this storyline of how you could kind of, sort of, basically clean up the sperm and, you know, get her pregnant. I’ll be honest, that’s more or less when I stopped watching… But my understanding is that… if I want to father a child, that in itself can be accomplished these days without passing on HIV to the child. (Paul, aged 45)

I remember quite vividly the whole storyline with Mark Fowler in *EastEnders*. And they kept on talking about how he was going to have a kid with his wife or girlfriend or whoever she was. And they talked about sperm washing, which just sounded utterly ridiculous but it sort of made me think that they can do something about it. (Richard, aged 39)

Social media was another source of information about sperm washing:

I saw someone on Facebook – a friend of a friend of a friend had a child through sperm washing. I think it’s a lesbian couple, not sure. And… yes, so, I just really didn’t know about that at all. It just seemed impossible, it seemed like science fiction. If I’d known about that, I could have imagined it. (Lewis, aged 32)

Sperm washing evidently captured men’s imagination. For Lewis, quoted above, his Facebook encounter with the technique revealed a possibility he had previously been unable to imagine – he talked about it as an opportunity he wished he had known about earlier. Another man did not seem to know about sperm washing, but he nevertheless imagined it as a future possibility:

Is it that one day we’ll be advanced enough to remove the virus from your sperm so that you can actually still be a parent and use it with a surrogate to have a kid? I think one day that might become a reality, very soon… Maybe they have successfully done that but whether they have commercialised it… maybe in the labs it’s possible now. It would be interesting to know actually – if somebody told me, you know what, we can remove HIV from the sperm and you can have a kid. That would be awesome. (Wei, aged 33)

As we have shown so far, many men in our study seemed to perceive the ability to have (biological) children as conditional upon the ability to remove HIV from their semen. Thinking about parenthood appeared to generate associations with the possibility to ‘clean up the sperm’, whether the men referred to sperm washing directly or only alluded to it by describing what they thought the technique involved. None of the men, however, had detailed knowledge about sperm washing and pondering it led some to question their understanding of HIV transmission. This contrasted with perspectives of healthcare practitioners.

### 4.2 ‘Hardly anyone these days pursues that sort of route’

Similar to men living with HIV, HIV clinicians also often referred to sperm washing unprompted – usually in relation to heterosexual men. However, unlike patients, who saw sperm washing as a present or future possibility, practitioners described it as a procedure that was used in the past:

When I started [working in HIV medicine 12 years ago], if you were a man with HIV and you wanted to get a woman pregnant, then you had to go to the Chelsea and Westminster fertility unit for sperm washing. (physician)

I remember the days when it was all terribly elaborate for heterosexual couples – you know, if one was positive and one was negative and they wanted to conceive – sperm washing and all of that stuff. (sexual health adviser)

[At that time], when positive men wanted to conceive, we were still referring for sperm washing… Hardly anyone these days pursues that sort of route. (physician)

Clinicians regarded sperm washing as ‘nearly unnecessary’ and ‘almost redundant’ because they universally recognised that HIV‐positive men who were on treatment and whose viral load was undetectable would not transmit the virus:

Undetectable viral load means you can have children without having to go through all these procedures. (psychologist)

If you’ve got an undetectable viral load and your partner knows your diagnosis, you can have unprotected sex. (nurse)

Most consultants, if they see that [the patient is] on antiretrovirals, undetectable and compliant with the medication, they tell them to just do it naturally. (nurse)

No practitioners said that there was ‘zero risk’ of HIV transmission, but they emphasised that the risk was ‘negligible’ and ‘probably non‐existent’. Some commented on the lack of awareness of how minimal the risk of transmission is – among the general public and even among healthcare professionals:

I really don’t think a lot of people would know that if you’re undetectable the risk of transmission is very, very small… I mean, even if you were, you know, a GP, would you be aware of that? You probably wouldn’t be. (physician)

It seems like everyone has an assumption that it isn’t possible to have a family [when you’re HIV‐positive]. And to be honest… before I worked in sexual health, I would have thought the same. (sexual health adviser)

According to clinicians, as these two quotations highlight, knowledge about what being undetectable means, and what implications it has for having a family, was so uncommon that it was possibly limited to professionals working in sexual health. Practitioners also noted that awareness among people living with HIV, although growing, was still low and patients had a tendency to overestimate the risk of transmission:

It just surprises me how little a lot of people still know about risks of, you know, vertical transmission. I mean, I still see ladies who have no idea what the risk of their baby acquiring HIV would be… And it’s really quite sad, you know. You can see that massive sort of relief when you’re actually telling them that, you know, these are possibilities. (physician)

One possible explanation for why even people who are HIV‐positive often ‘have no idea’ about the small risk of their child acquiring HIV could be that they remain uninformed about the concept of an undetectable viral load. However, in our interviews with patients, it was evident that undetectability had become an established part of their vocabulary – in fact, it was central to how the men talked about living with HIV. Of the 23 gay men, none of whom were asked about it directly, 18 used the word ‘undetectable’ at some point in the interview – usually in relation to their own viral load or when describing themselves. In most cases, they did so when talking about sex, highlighting the difference becoming undetectable had made to their intimate lives. Similar to the clinicians quoted earlier, the men did not suggest that having an undetectable viral load meant no risk of HIV transmission but, as one of them put it, it was ‘incredibly, incredibly hard’ to pass HIV on:

I’m on my medication, I’m undetectable, so I’m virtually – not entirely, but virtually uninfectious. So I think that it doesn’t really affect my life now. It’s not something that bothers me. (Ben, aged 33)

I think, you know, being on treatment and being told, well, your viral load is undetectable, while I was in a relationship and, actually, you know, the chances of me passing this on are much reduced – that, I think, psychologically, for me, was sort of… it just sort of felt like everything was back on an even keel and sort of, like, okay. (Rory, aged 35)

For men like Rory, quoted above, finding out that their viral load was undetectable was a turning point: it reduced not only the risk of ‘passing this on’, but also the role HIV played in their lives. However, even though the men often commented on how important being undetectable was for their sexual relationships, they rarely mentioned undetectability as something that might be relevant in relation to parenthood. In fact, only two men made an explicit connection between sexual and reproductive relationships when talking about the implications of being undetectable. Peter, aged 35, recalled being told by his doctor about how antiretroviral treatment prevented HIV transmission:

I didn’t actually know that once you are on treatment and, you know, you’re undetectable, then, you know, you can’t, you’re not going to pass on HIV, not even to a child. And so, yes, so that was new information for me. So up until then, I guess I thought that, yes, I’m now disqualified.

Lucas, aged 42, was the other man who mentioned undetectability in relation to parenthood, though only after referring to sperm washing:

I think I’ve read about techniques, about people washing your sperm and being able to select the ones that are free from the virus. And I’m 100% undetectable. You know, I shouldn’t be saying that, but I have unprotected sex with [my partner] because he prefers it. And he never converted in all these years. So I think it’s very possible that I can father children and they won’t have HIV. And I think there’ll be a lot of people as well that maybe through technology or through other things can father children. So I don’t think that, in the near future, or maybe right now, it would be an issue, you know.

Peter and Lucas were the only men who seemed to recognise an opportunity for HIV‐positive men to become parents not only in assisted reproductive technology such as sperm washing, but also in antiretroviral therapy. For most men, being undetectable did not seem to signal parenthood possibilities, which was reflected in how they talked about different pathways to parenthood.

### 4.3 ‘Would I transmit HIV to the baby?’

In our interviews, men living with HIV talked about various ways of having children or enabling others to become parents, including surrogacy, adoption and sperm donation. Some men suggested that being HIV‐positive made them unsuitable to provide semen for female friends who had asked them in the past about being a sperm donor:

I’ve always had close girlfriends – which is obviously a bit unfortunate given where I’m aiming at – but I’ve always had these girls that aren’t, like, in love and I would sort of joke and say, oh well, you know, if you get to 35 and you haven’t got a kid, I’ll give you one… But obviously you’ve then got this problem because you can’t actually give someone this healthy baby. (Paul, aged 45)

Lesbian friends of mine, they’re like, oh, would you be my sperm donor? That was always a bit of a joke, you know. But then, when these questions arose once I was diagnosed with HIV, it was like, oh, I don’t think you want that. (Peter, aged 35)

Other men excluded the possibility of biological parenthood and presented adoption as ‘the only option’:

One day me and my partner talked about, like, if you didn’t have HIV, you could have your child with a woman and – you know, you could give your own sperm and have a child. But because we are both HIV‐positive we don’t have that option. So the only way for us is to adopt a child. (Frank, aged 27)

Well, I know it can’t be my own specimen because it’s infected. But adoption – yes. Giving somebody an opportunity for a better life – I think it’s a beautiful thing to do. (Anton, aged 29)

Some men were unsure whether surrogacy was an option for HIV‐positive men and implied that their HIV status might be one complication too many for what is already a complex procedure. For example, 29‐year‐old Lee reflected on how becoming HIV‐positive had made him rethink his attachment to the idea of having biological children:

I always imagined maybe I would do, like, surrogacy or something. And then I realised, well, [being HIV‐positive] might affect that – because I don’t even know how that would work now given my status. Would I transmit HIV to the baby? I knew that they could do things to stop it, but would that make the whole process even more expensive? And then, at that point, I thought, how much of an inconvenience am I causing just so that I can have a genetically, biologically related child? And is that really even that important to me?

The concern about transmitting HIV in surrogacy was echoed by 32‐year‐old Lewis who had direct experience with a US‐based surrogacy agency and a more detailed understanding of the implications of being HIV‐positive for finding a surrogate. He recalled a conversation with the agency, which had told him and his partner about clients who had used sperm washing in the past:

We were told they’d had situations where they said, it’s great, your sperm is clean, and the surrogate goes, no, I’m not doing it… It’s just, you know, a human factor, people get scared… They told us about a surrogate who just freaked out, because she read [the surrogacy contract] the way it’s written – supposedly it’s just very legalistic and very worst‐case scenario, you know, as in, you cannot sue us if you become HIV‐positive… They were just like, you know, the sperm may be wonderful but in real life the surrogate balks and they freak out at the very last moment. They said, we don’t want you going through all that stuff to only find that out. In theory, it’s a possibility, we could go through the whole thing and it could be totally fine. But in practice it’s very unlikely.

As this quotation illustrates, it was not so much the risk of HIV transmission but the risk of a potential surrogate changing her mind that stood in the way of Lewis pursuing surrogacy with the use of his own sperm. The surrogacy agency had used sperm washing in the past, which ensured that there was *no risk* (as opposed to minimal risk) of HIV infection, but a clause in the contract, protecting the agency from any liability, conveyed a different message: it implied that some risk was still there.

From what we have seen so far, it seems that imagining biological parenthood for men living with HIV depends on shifting the perception of risk from negligible to non‐existent. But some men suggested that even reducing the risk of transmission to zero could still be insufficient to consider having biological children as an actual option. When thinking about the possibility of becoming a father via surrogacy, 33‐year‐old Wei was concerned about HIV infection, even if the risk of transmission could be eliminated:

First of all, how safe is it for the kid? Would you pass on the virus to the kid? And would you pass on the virus to the host, whoever carried the kid? And at the moment I don’t think anybody would be able to do that. If someone said, oh, would you mind carrying a baby for me – but, by the way, I’m HIV‐positive – they’ll go, whoa, whoa, no, maybe not. If I were the person who was asked that question, I don’t think I’d say yes – I’d definitely say no… Even if the doctor said, alright, this patient, he wants to have a kid, we’ll remove the virus but we have to let you know that he’s HIV‐positive – I think people would still have a problem accepting it. At least I would.

To sum up, when describing different routes to parenthood, men in our study were highly concerned about the risk of transmitting HIV to a child or to the woman with whom the child would be conceived. Assuming that they could not conceive a ‘healthy baby’ because their ‘own specimen’ was ‘infected’, some men completely ruled out the possibility of biological fatherhood, seeing adoption as ‘the only way’ to have children. For some, even if HIV transmission risk was eliminated, sperm donation or surrogacy was difficult to imagine. But why, we may ask, were concerns about transmission so strong when the men saw themselves as ‘undetectable’? Put another way, why would men who were reassured about their undetectable viral loads to the extent they no longer worried about the risk of HIV transmission when having sex be so vigilant about transmission risk when it came to having children?

## 5 DISCUSSION

As our interview data illuminate, for HIV‐positive gay men, imagining parenthood is full of uncertainties and assumptions. Many men we spoke with excluded the possibility of having children in the future, even when parenthood seemed otherwise desirable, because of preconceptions about HIV transmission. But these preconceptions also indirectly affected men who were not interested in having children. Even when ideas about what living with HIV meant for parenthood seemed irrelevant, such assumptions seemed to play a role in how men thought about themselves. Our findings suggest that, irrespective of parenting desire, a perceived inability to have children due to being HIV‐positive can influence men’s overall self‐perception. In particular, despite an apparent awareness of what having an undetectable viral load means for sexual relationships, the incomplete understanding of HIV transmission can maintain a perception of oneself as infectious, which can perpetuate internalised stigma.

In our interviews, we identified a stark contrast in how gay men living with HIV and HIV clinicians talked about the possibility for HIV‐positive men to become parents. Whereas the men regarded sperm washing as a present or even future possibility, clinicians described it as a procedure used in the past and no longer necessary. At the same time, while clinicians highlighted the implications of being undetectable for HIV‐positive men’s parenting desires and intentions, the men usually talked about undetectability only in relation to sexual relationships. This finding highlights a discrepancy in patients’ and practitioners’ perceptions of what options exist for HIV‐positive men who want to become parents. In the context of rapidly changing HIV science, it is not necessarily surprising that patients’ knowledge does not reflect current medical knowledge. Previous research has shown that gay men’s understandings of undetectability in relation to sexual relationships are often more conservative than those based on most recent clinical research (Bourne *et al*. 2016, Grace *et al*. 2015). We might expect that as knowledge about U = U becomes more mainstream, the awareness of parenthood possibilities among gay men will also increase, as it seems to have done in relation to their sex lives.

Whereas the striking contrast between patients and practitioners in how they talked about sperm washing and being undetectable is significant in itself, what is especially revealing is our finding that HIV‐positive gay men associated assisted reproductive technology (removing the virus from semen), rather than antiretroviral therapy (reducing the viral load), with the possibility of safe conception and hence parenthood. Considering how important being undetectable was for the men’s sex lives, and how being undetectable has become a central aspect of identity for many HIV‐positive gay men (Grace *et al*. 2015, Race 2015), it is perhaps surprising that it was not the potential of viral undetectability that opened up reproductive possibilities in the men’s imagination. Instead, what seemed to capture their imagination was a technique which they had only heard about in passing. As such, by showing how (and what kind of) biomedical knowledge is incorporated into the understanding of oneself and one’s body as HIV‐positive, our findings shed further light on the question of whether, in the TasP era, HIV can be ‘reimagined’ (Persson 2013, 2016). It seems that people’s perception of their body as contagious can be so ingrained that even knowing that you ‘can’t pass it on’ can be insufficient to consider oneself as non‐infectious.

The interviews this article draws upon were conducted between May and December 2016, and it is important to interpret our findings in the context of the timing of data collection. In particular, it is useful to keep in mind that the U = U campaign launched in early 2016 but it was not until the second half of 2017 when the campaign was officially endorsed by the British HIV Association and public health organisations worldwide such as the US Centers for Disease Control and Prevention. This explains why practitioners interviewed for our study talked about the implications of having an undetectable viral load with some reservation. They did not say that being undetectable entailed *zero risk* of HIV transmission to sexual partners. Instead, they described transmission risk as ‘negligible’, ‘probably non‐existent’ or ‘very, very small’, which made them deem sperm washing as ‘nearly unnecessary’ and ‘almost redundant’. This linguistic caution reflects the dominant clinical position at the time of our interviews and we would expect that a different language would have been adopted had the data been collected 1 or 2 years later. Likewise, although we can only hypothesise, it is also possible that, for men living with HIV, being undetectable in the current climate – when there are concerted efforts by HIV organisations to increase public awareness of U = U – would be even more at the forefront of their minds, which could prompt more extensive thinking about the implications of undetectability. However, perceptions of risk, or lack thereof, are complex, as they are imbued with emotions, moral judgements and a variety of sociocultural factors (Lupton 2013). As such, the rational logic of U = U may encounter affective barriers, making people living with HIV likely to exaggerate the risk of transmission, even when they are told that the risk is not only reduced but eliminated.

As reliable knowledge about the risk of sexual HIV transmission is currently disseminated in unambiguous messages, it is worth considering how we can move beyond ‘strictly sexual’ understandings of undetectability so that a similar consensus can be reached about the risk of non‐sexual HIV transmission. For example, as Waitt *et al*. (2018) argue, there is currently insufficient evidence to state with certainty that undetectable equals untransmittable in the context of breastfeeding. Further research should examine what ways of communicating about risk are appropriate at a time when there is scientific certainty about U = U in the context of sexual HIV transmission but when science might be lacking to provide a similarly clear message about HIV transmission in other contexts. In addition to clinical evidence, what is needed is further research on perceptions of risk in sexual and reproductive relationships, especially studies that consider both from a comparative perspective, so that we can better understand the logics behind people’s assumptions about infectiousness. Paying attention to different bodily fluids, such as blood, semen and breastmilk, may offer valuable insights for understanding these logics.

One issue to keep in mind is that the role of risk in the context of our study is, quite possibly, not limited to the potential of HIV infection. In other words, the overemphasis on risk evident in the narratives of men living with HIV may result not only from an exaggerated perception of their infectivity. It may also reflect a broader cultural trend towards *anticipating risk*, which is especially apparent when it comes to reproduction (Waggoner 2017). Even if HIV science continues to demonstrate that U = U in a variety of non‐sexual contexts, the translation of the ‘zero risk’ message to the reproductive domain can encounter not only affective barriers but also competing discourses of risk.^3^ People considering parenthood are likely to be aware of an ever‐increasing multiplicity of reproductive risks. If anything, rather than feeling increasingly reassured about safety, they are likely to approach the prospect of having children with growing anxiety (Faircloth and Gürtin 2018). Besides, for men such as interviewees in our study, parenthood‐related anxiety may not always be countered by parenting desire: with some exceptions, even among men who wanted to have children in the future, the desire to conceive children with their own sperm did not seem strong enough to be ‘worth’ the risk. While it is important to reinforce the U = U message with further evidence, it is likely that, in the reproductive realm, exaggerated awareness of risk will endure.

The findings of our study have specific implications for HIV care. For a start, it is important to recognise that HIV‐positive gay men also have parenting desires, and not assume that knowledge about parenthood is only relevant to heterosexual people. This knowledge, as our findings show, can have beneficial effects also on those who do not want to have children, since realising that parenthood is a possibility can foster self‐esteem and a more optimistic attitude to life. In addition, clinicians and other professionals who work with people living with HIV should be aware that many gay men perceive sperm washing as the go‐to method for HIV‐positive men who want to pursue biological fatherhood. It is important to consider how this perception might affect patients’ understandings not only of their own reproductive possibilities but also of the mechanics of HIV transmission. Even when patients demonstrate a good understanding of what it means to be undetectable for their sexual relationships, the implications of being undetectable for reproduction can be less well understood.

Notwithstanding the complexity of risk, and the potential specificity of reproductive risk, our findings suggest that it is misconceptions about HIV transmission that constitute the main barrier for gay men living with HIV to imagine biological parenthood as a possibility. The well‐documented persistence of HIV stigma (Jaspal and Nerlich 2020, Rai *et al*. 2018, Walker 2019) undoubtedly plays a critical role in the maintenance of these misconceptions. To reduce the stigma and minimise the extent to which HIV‐positive people internalise it, there is a continuing need to communicate that *undetectable equals untransmittable*, as recent studies on U = U highlight (Grace *et al*. 2020, Okoli *et al*. 2020, Rendina *et al*. 2020). Paying more attention to aspects of same‐sex intimacy other than sexual relationships might help achieve this goal.

## AUTHOR CONTRIBUTIONS

**Robert Pralat:** Conceptualization (lead); data curation (lead); formal analysis (lead); funding acquisition (lead); investigation (lead); methodology (lead); project administration (lead); writing – original draft (lead); writing – review and editing (lead). **Fiona Burns:** Conceptualization (supporting); investigation (supporting); methodology (supporting); writing – review and editing (supporting). **Jane Anderson:** Conceptualization (supporting); investigation (supporting); methodology (supporting); writing – review and editing (supporting). **Tristan J. Barber:** Conceptualization (supporting); investigation (supporting); methodology (supporting); writing – review and editing (supporting).

## FUNDING

This work was supported by the British HIV Association, the Wellcome Trust (grant number 100606/Z/12/Z), the Leverhulme Trust (grant number ECF‐2018‐146) and the Isaac Newton Trust. We are grateful to all the funders for their generous support.

## Data Availability

Data are not publicly available due to ethical restrictions and concerns about protecting interviewees' anonymity.

## ENDNOTES

1 Although our original intention was to capture views and experiences of both gay and bisexual men, only one man who identified as bisexual took part in our study, which meant that we were unable to represent bisexual perspectives in a meaningful way. We therefore describe men who are the focus of this article as gay men, while remaining attentive to potential relevance of our findings to bisexual and other men who have sex with men.

2 An HIV viral load, measured in copies per mL of blood, is undetectable when it cannot be detected by standard viral load testing.

3 We are grateful to one of the anonymous reviewers for drawing our attention to this issue.
